# Do Anxiety and Depression Predict Persistent Physical Symptoms After a Severe COVID-19 Episode? A Prospective Study

**DOI:** 10.3389/fpsyt.2021.757685

**Published:** 2021-11-10

**Authors:** Hugo Bottemanne, Clément Gouraud, Jean-Sébastien Hulot, Anne Blanchard, Brigitte Ranque, Khadija Lahlou-Laforêt, Frédéric Limosin, Sven Günther, David Lebeaux, Cédric Lemogne

**Affiliations:** ^1^Paris Brain Institute - Institut du Cerveau et de la Moelle Épiniére, UMR 7225, UMR_S 1127, CNRS, INSERM, Sorbonne University, Service de Psychiatrie de l'Adulte, Hôpital de la Pitié-Salpêtriére, DMU Neurosciences, Assistance Publique-Hôpitaux De Paris, Paris, France; ^2^Service de Psychiatrie de l'Adulte, DMU Psychiatrie et Addictologie, Hôpital Hôtel-Dieu, Université de Paris, Assistance Publique-Hopitaux de Paris, Paris, Paris, France; ^3^CIC 1418 and DMU CARTE, Assistance Publique Hopitaux De Paris, Hôpital Européen Georges-Pompidou, Paris, France; ^4^Service de Néphrologie, Assistance Publique Hopitaux De Paris, Hôpital Européen-Georges Pompidou, Université de Paris, Paris, France; ^5^Service de Médecine Interne, Assistance Publique Hopitaux De Paris, Hôpital Européen-Georges Pompidou, Université de Paris, Paris, France; ^6^DMU Psychiatrie et Addictologie, Service de Psychiatrie de l'Adulte, Assistance Publique Hopitaux De Paris, Hôpital Européen-Georges Pompidou, Université de Paris, Paris, France; ^7^DMU Psychiatrie et Addictologie, Service de Psychiatrie de l'Adulte, Assistance Publique Hopitaux De Paris, Hôpital Corentin Celton, Université de Paris, Paris, France; ^8^Innovative Therapies in Haemostasis, INSERM, Université de Paris, Paris, France; ^9^Service de Physiologie, Assistance Publique Hopitaux De Paris, Georges Pompidou European Hospital, Paris, France; ^10^Service de Microbiologie, Unité Mobile d'Infectiologie, Assistance Publique Hopitaux De Paris, Hôpital Européen Georges Pompidou, Université de Paris, Paris, France; ^11^Service de Psychiatrie de l'Adulte, Hôpital Hôtel-Dieu, DMU Psychiatrie et Addictologie, Assistance Publique Hopitaux De Paris, INSERM, Institut de Psychiatrie et Neurosciences de Paris, UMR_S1266, Université de Paris, Paris, France

**Keywords:** anxiety, COVID-19, long Covid, post-covid condition, post-acute COVID-19 syndrome, somatoform disorders, depression, fatigue pain

## Abstract

**Background:** Persistent physical symptoms are common after a coronavirus disease 2019 (COVID-19) episode, but their pathophysiological mechanisms remain poorly understood. In this study, we aimed to explore the association between anxiety and depression at 1-month after acute infection and the presence of fatigue, dyspnea, and pain complaints at 3-month follow-up.

**Methods:** We conducted a prospective study in patients previously hospitalized for COVID-19 followed up for 3 months. The Hospital Anxiety and Depression Scale (HAD-S) was administered by physicians at 1-month follow-up, and the presence of fatigue, dyspnea, and pain complaints was assessed at both 1 month and 3 months. Multivariable logistic regressions explored the association between anxiety and depression subscores and the persistence of each of the physical symptom at 3 months.

**Results:** A total of 84 patients were included in this study (Median age: 60 years, interquartile range: 50.5–67.5 years, 23 women). We did not find any significant interaction between anxiety and the presence of fatigue, dyspnea, or pain complaints at 1 month in predicting the persistence of these symptoms at 3 months (all *p* ≥ 0.36). In contrast, depression significantly interacted with the presence of pain at 1 month in predicting the persistence of pain at 3 months (OR: 1.60, 95% CI: 1.02–2.51, *p* = 0.039), with a similar trend for dyspnea (OR: 1.51, 95% CI: 0.99–2.28, *p* = 0.052).

**Discussion and Conclusion:** Contrary to anxiety, depression after an acute COVID-19 episode may be associated with and increased risk of some persistent physical symptoms, including pain and dyspnea.

## Introduction

The coronavirus disease 2019 (COVID-19), caused by the novel severe acute respiratory syndrome coronavirus 2 (SARS-CoV-2), has widely spread worldwide (1). Approximately 20% of infected individuals develop severe illness, requiring hospitalization, but most experience mild symptoms, and did not beneficiate for a long-lasting follow-up (2). Among these patients, a substantial number still present clinical symptoms at a significant distance from acute infection (3–6). A meta-analysis including 1,816 hospitalized patients found that fatigue, dyspnea, chest pain, and cough, were the most prevalent persistent symptoms in 52, 37, 16, and 14% of survivors between 3 weeks and 3 months after hospitalization, respectively (6). These persistent physical symptoms following COVID-19 are now referred to as “Long COVID,” or “Post-COVID syndrome.” However, it is noteworthy that they may not be specific to SARS-CoV-2 infection (7). Indeed, although the prevalence of these persistent symptoms is now better known, little is known about their pathophysiological mechanisms as well as the potential clinical predictive factors that could be used for prevention strategies. This question could therefore represent a major public health concern over the next few years, and supports the urgent need to understand the pathophysiological mechanisms and clinical predictors of these symptoms (8, 9).

Several hypotheses have been formulated to explain these persistent physical symptoms, such as the impact of direct effects of the virus, sequelae of neurological damage, persistent inflammatory syndrome, or involvement of cognitive mechanisms akin to those observed in functional somatic disorders (8). Severe acute respiratory syndrome coronavirus 2 infection produce inflammatory disturbing, up to a so-called “cytokine storm” (i.e., a systemic production of cytokines, chemokines, and inflammatory mediators) (10). This immune-inflammatory activation may induce thromboses and vascular damages, and disturb hypothalamo-pituitary-adrenal and neuroendocrine axes (11). In addition to these causes, psychiatric symptoms could be one of the keys in understanding these persistent physical symptoms (12, 13).

Psychiatric symptoms are frequently found after a coronavirus infection such as SARS-CoV-1 or MERS-CoV (14–16). Supporting these findings, current reports showed that SARS-CoV-2 infection is associated with a high prevalence of anxiety and depression afterwards (17–19). Persistent and disabling symptoms could induce depressive symptoms and anxiety (20–22) and persistent physical symptoms after COVID-19, including gastrointestinal and respiratory symptoms, are significantly associated with a higher probability of developing psychiatric symptoms (19, 23, 24). Reciprocally, however, patients presenting with depression and anxiety also tend to have more somatic and pain complaints (25). Anxiety and depression have been identified as highly associated with persistent symptoms in several conditions (26), including fatigue after COVID-19 (27). Furthermore, among the most frequent persistent symptoms after COVID-19, many are either core diagnostic criteria for depressive or anxiety disorders (i.e., fatigue, sleep disorders, cognitive disturbances) or symptoms frequently associated with these disorders (e.g., dyspnea and panic disorder, pain and depressive disorders). For instance, a study from our group found that cognitive complaints at 1 month after follow-up were better explained by the levels of anxiety and depression than by objective neuropsychological functioning (28). The high prevalence of anxiety and depression symptoms after COVID-19 may thus partially account for some persistent symptoms. Nevertheless, this hypothesis may have been somewhat overlooked so far, despite clear clinical implications, as evidence-based treatments are available for both depression and anxiety.

In this preliminary study, we aimed to explore the association between anxiety and depression 1 month after hospitalization for acute SARS-Cov-2 infection and the presence of persistent physical symptoms (fatigue, dyspnea, and pain complaints) after 3 months. Specifically, we thought that persistent physical symptoms would particularly occur in patients presenting both a physical symptom and manifestations of anxiety and depression. First, the presence of both at 1 month could indicate that the persistent physical symptom is potentially a psychiatric symptom. Second, anxiety and depression may share some vulnerability factors with functional somatic symptoms (e.g., female gender, high level of neuroticism) and thus be associated with an increased risk of persistent physical symptoms after an acute COVID-19. In other words, we hypothesized that anxiety and depression at 1 month would predict persistent physical symptoms at 3 months for patients presenting these symptoms at 1 month.

## Materials and Methods

### Population

The study is part of The French COVID cohort (NCT04262921) that was authorized by the French Ethics Committee (ID RCB:2020-A00256-33). All inpatients aged 18 or more who were hospitalized at European Hospital Georges Pompidou for a SARS-CoV-2 infection (according to WHO criteria) during the first wave of the COVID-19 pandemic, between March 17th and April 29th, 2020 were included. Participants underwent a standardized physical evaluation by a senior physician at the time of the inclusion (i.e., within the first 48 h of hospital admission for COVID-19). Socio-demographic data as well as clinical and biological data regarding the infection were recorded at inclusion, using a standardized medical form. Clinical data included admission in intensive care unit (ICU) as a proxy of severity of the infection. Written informed consent was obtained for all participants.

Patients who were discharged less than a month after the hospitalization for COVID-19 were proposed a clinical follow-up at 1 month, 3 months, and 6 months, either during a 1-day outpatient clinical examination or by teleconsultation. The present study is based on data collected during 1-day outpatient clinical examinations at 1 month and 3 months.

### Clinical Assessment

To assess anxiety and depression symptoms, the Hospital Anxiety and Depression Scale (HAD-S) was filled at 1-month follow-up. The HAD-S encompasses 14 items coded 0–3 and two subscales (HADS-A: seven items measuring symptoms of anxiety; HADS-D: seven items measuring symptoms of depression). Several studies have shown valid psychometric properties for HAD-S subscales in the general population (29), in patients suffering from various pathologies (30), for older patients aged 65–80 years (31), and in different countries (32). In the present study, the Cronbach's alpha coefficient for the global score, the anxiety subscore and the depression subscore was 0.86, 0.84, and 0.72, respectively, suggesting satisfactory internal consistency.

Patients also underwent a clinical examination at 1- and 3-month follow-up that included a general physical examination with systematic assessment of the following residual symptoms: agueusia, anosmia, cough, dyspnea, myalgia, arthralgia, fatigue, headache, rhinorrhea, diarrhea, nausea. Here, we focused on fatigue, dyspnea, and pain complaints (defined as presenting with arthralgia, myalgia, or headache) that were self-reported. Clinical examinations were performed by a team of physicians working in the same hospital, but each patient was not necessarily examined by the same clinician.

The presence of a restrictive ventilatory defect, defined as having a total lung capacity <80% at spirometry test at 1-month follow-up was also recorded, as an alternative proxy of severity of the infection. Patients also had biological investigations including measure of the blood level of C-reactive protein (CRP), an inflammatory marker, at 1-month.

**Figure 1:**
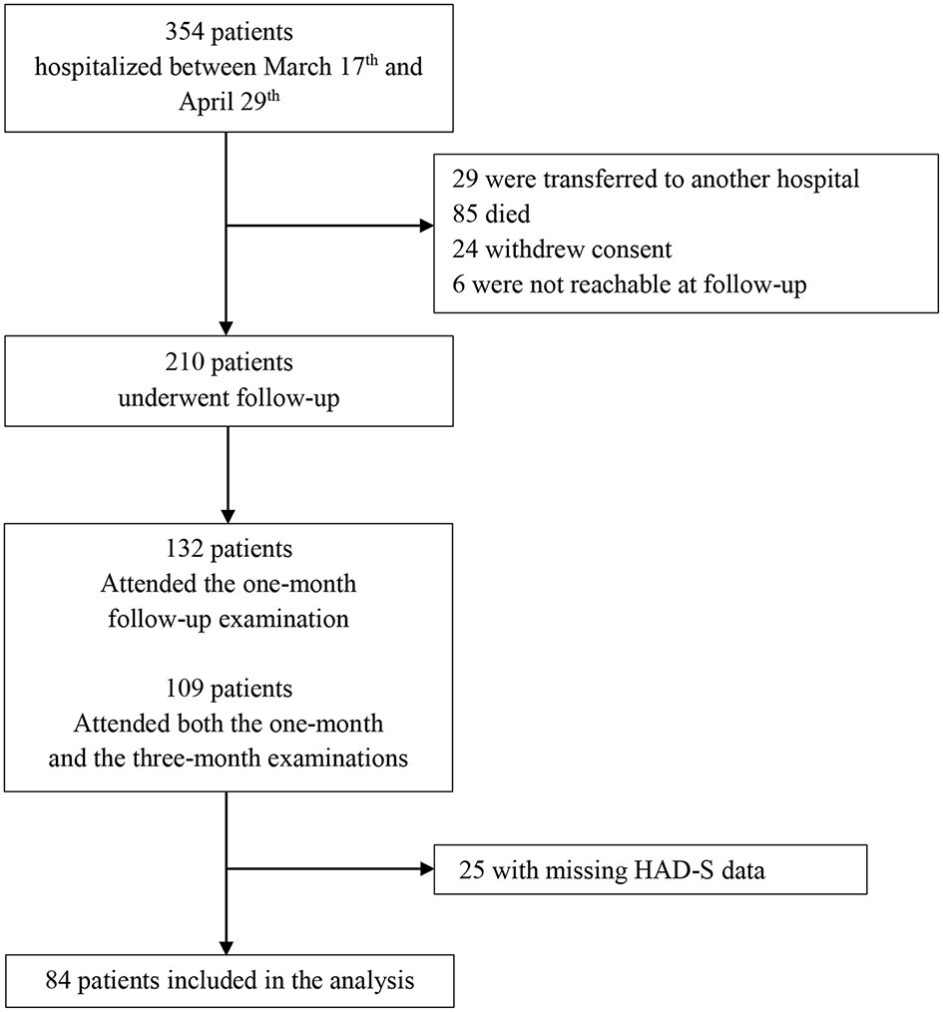
Flow of patients in the study. Between March 17th and April 29th, 2020, 354 patients hospitalized at HEGP were included in the French COVID cohort. Among these patients, 29 were lost at follow-up because of inter-hospital transfer, 85 died, 24 withdrew their consent, and 6 were not reachable. Among the 210 remaining patients who underwent the follow-up, 132 attended the 1-day follow-up examination at 1 month, including 109 who also attended the 1-day follow-up examination at 3 months. Among these 109 patients, 84 had HAD-S data at 1-month follow-up.

Between March 17^th^ and April 29^th^, 2020, 354 patients hospitalized at HEGP were included in the French COVID cohort. Among these patients, 29 were lost at follow-up because of inter-hospital transfer, 78 died and 2 withdrew their consent. Among the 245 patients eligible for the clinical follow-up, 7 died during the follow-up, 22 withdrew their consent, and 6 were not reachable. Among the 210 remaining patients who underwent the follow-up, 132 attended the 1-day follow-up examination at 1 month, including 109 who also attended the 1-day follow-up examination at 3 months. Among these 109 patients, 84 had HAD-S data at 1-month follow-up. Figure 1 presents the flow of the study participants.

### Statistical Analysis

Descriptive analyses were performed for the categorical and continuous variables of interest.

For each physical symptom considered (i.e., fatigue, dyspnea, and pain complaints), two different binary logistic regression models were built for the two dimensions of the HAD-S (Anxiety and Depression), as some symptoms may be particularly in link with one of those dimension (e.g., fatigue or pain complaints with depression). The outcome was the presence of the physical symptom at 3 months and the predictive variables were its presence at 1 month and one of the HAD-S subscores. Furthermore, to test our hypothesis, every binary logistic regression models included an interaction term between the presence of the physical symptom at 1 month and the HAD-S subscore. All models were also adjusted for age, sex, and admission in ICU (as a clinical marker of the initial severity of the infection).

We ran sensitivity analyses to test the robustness of our results: admission in ICU was successively replaced with the presence of a restrictive ventilatory defect at 1 month, the blood level of CRP at 1 month (log-transformed), and the presence of a medical comorbidity (i.e., diabetes or high blood pressure) at 1 month. The significance threshold was *p* < 0.05. All analyses were performed using Stata 15.0 (StataCorp, College Station, TX).

## Results

The characteristics of the 84 participants [23 women, median age: 60 (interquartile range (iQr): 50.5–67.5] are displayed in Table 1.

**Table 1:** Participants' characteristics (*n* = 84).

**Continuous variables**	**Median**	**iQr**
Age	60	50.5–67.5
HAD-S anxiety score	6	4–8
HAD-S depression score	4	2–6.5
**Categorical variables**	***N***	**%**
**Sex**		
Men	61	72.6
Women	23	27.4
ICU admission	26	31.0
Restrictive ventilatory defect^*^ at 1-month follow-up (*n* = 80)	24	30.0
**Comorbidity**		
High blood pressure	31	36.9
Diabetes	17	20.2
Chronic cardiac disease	10	11.9
Asthma	11	13.1
Other chronic pulmonary disease	6	7.1
Renal disease	7	8.3
**Physical symptom at 1-month follow-up**		
Fatigue	50	59.5
Dyspnea	50	59.5
Pain complaints (*n* = 83)	29	35.4
**Physical symptom at 3-month follow-up**		
Fatigue (*n* = 82)	38	46.3
Dyspnea	39	46.4
Pain complaints (*n* = 83)	26	31.3

* *Presence of a Restrictive ventilatory defect was defined as having a total lung capacity <80% at spirometry test*.

Table 2 displays the results of the six different multivariable logistic regressions models searching for an interaction between each of the physical symptoms considered (fatigue, dyspnea, and pain complaints) and each of the HAD-S subscore (anxiety and depression) at 1 month in predicting the persistence of the symptom at 3 months.

**Table 2:**
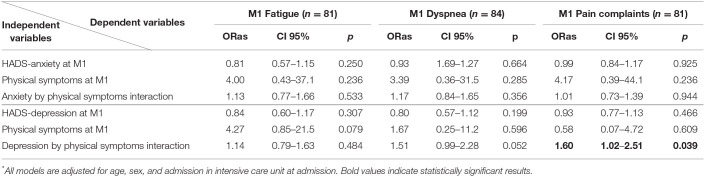
Factors associated with fatigue (model 1), dyspnea (model 2), and pain complaints (model 3) at 3-month follow-up in multivariable ^*^ logistic regression.

For anxiety, we did not find any significant interaction between anxiety and fatigue, dyspnea, or pain complaints at 1 month in predicting the persistence of any of these symptoms at 3 months. For depression, we found a significant interaction with the presence of pain at 1 month in predicting the persistence of pain at 3 months, with a similar, yet not significant trend for dyspnea (Table 2). Concerning the other variables included in the regression models, none of them were significantly associated with the presence of any symptom at 3 months.

Replacing “admission in ICU” with another variable measuring the severity of the infection or possible sequelae (i.e., level of CRP at 1-month follow-up or having a restrictive ventilatory defect at 1-month follow-up), or comorbid conditions associated with more severe infection (i.e., diabetes or high blood pressure) did not change the observed associations or size effects. The depression by pain complaints interaction remained significant [adjusted OR [confidence interval (CI)]: 1.78 [1.05–3.02], 1.61 [1.01–2.55], 1.60 [1.02–2.51], and 1.68 [1.04–2.72], when adjusting for restrictive ventilatory defect, CRP level, comorbid diabetes, and comorbid high blood pressure, respectively], while the observed trend for the depression by dyspnea interaction even reached statistical significance for the sensitivity analysis adjusted for restrictive ventilatory defect, comorbid diabetes, and comorbid high blood pressure (adjusted OR [95% CI]: 1.57 [1.01–2.44], 1.57 [1.02–2.41], and 1.51 [1.01–2.25], respectively].

## Discussion

In our study, we explored the associations between anxiety and depression at 1 month after hospitalization for COVID-19 and persistent physical symptoms at 3 months. Although we did not find any evidence that anxiety may predict the persistence of fatigue, dyspnea, and pain complaints, there was a significant interaction between depression and pain complaints in predicting the persistence of those complaints, and a similar trend between depression and dyspnea.

We specifically found that the interaction between presenting at 1 month with pain complaints and greater HAD-S depression subscore was significantly associated with persisting pain complaints at 3 months. This latter result, indicating that patients with both a pain complaint and greater depressive symptoms at 1 month were more likely to present with persisting pain complaints at 3 months, is in line with previous and well-known findings, linking depression with more frequent pain complaints on one hand (12) and the persistence of physical symptoms after an acute infectious episode on the other hand (26). Although the lack of interaction with fatigue is at odd with recent findings suggesting that a history of depression may predict persistent fatigue in patients with COVID-19 (27), these discrepancies may be explained by the fact that current depression symptoms and history of depression are two different measures. In addition, it is noteworthy that our analyzes focused on the persistence of physical symptoms at 3 months rather than on the presence of these symptoms at 1 month. Interestingly, depression alone or pain alone at 1 month did not predict pain at 3 months. First, depression is frequently, yet not systematically associated with pain. Therefore, depression without pain at 1 month may indicate that there is no link between these two dimensions in a given patient, thus explaining why depression alone at month may not predict pain at 3 months. Second, pain without depression at 1 month may be associated with a more favorable course, for instance because of more adaptive health behaviors (e.g., higher levels of physical activity), than pain with depression, explaining why pain alone at month may not predict pain at 3 months.

Furthermore, our results approached statistical significance regarding dyspnea, in line with previous findings (13). Given the limited statistical power, our results are intriguing and should be the subject of further investigations. Several hypotheses could be explored regarding mechanisms accounting for the association between persistent painful complaints and depression after a severe COVID-19 episode. In particular, these persistent physical symptoms might be experienced more intensely by depressed patients, or these dimensions might involve shared factors such as a high level of neuroticism (i.e., tendency to experience negative emotions such as anger, fear, or sadness with limited tolerance for aversive stimuli) (33, 34). Noteworthy, our cohort may not be representative of the patients diagnosed with “long COVID” who are mostly young women with mild initial infection, so our results may not be generalized to these patients. Further studies should explore the role of these psychological dimensions regarding persistent physical symptoms after mild and severe COVID-19 episodes.

Regarding anxiety, our results do not suggest that general anxiety is predictive of persistent physical symptoms in COVID-19 survivors. It could be argued that having been included in a research cohort with planned follow-up may have reduced the level of anxiety, leading to underestimation of this parameter. Nevertheless, one should notice that the hypothesis of functional somatic disorder, which is defined by symptoms being insufficiently explained by impairment of the organs that they designate (e.g., dyspnea with no respiratory impairment) is not called into question by these results on general anxiety. Indeed, among the different cognitive and behavioral mechanisms involved in functional somatic disorders, illness attribution, and catastrophic beliefs, which may influence the perception of bodily perceptions, and behavioral avoidance (including physical activity cessation), which may influence the course of these symptoms, constitute two major factors (35) that are not assessed by the HAD-S depression and anxiety subscales. Among the available tools, the somatic symptom disorder-B criteria scale [SSD-12, (36)] is a validated scale that accurately explore the specific psychological features associated with functional somatic disorders. Future studies would benefit from specific assessment of these cognitive and behavioral mechanisms.

Some strengths of our study should be underlined. We used a prospective design with a 3-month follow-up of COVID19 survivors and provide an original approach in exploring the association between anxiety or depression and persistent physical symptoms within COVID-19 survivors. This prospective design allowed us to explore the presence of predictive factors, previously and independently assessed from the clinical outcomes. We also used HAD-S, a valid and reliable scale, which does not include an assessment of physical symptoms (37): we could thus distinguish the psychiatric symptoms evaluated by our scale, and the physical symptoms evaluated during a clinical interview.

However, our study also has some limitations: the monocentric recruitment of hospitalized patients only and the selection biases that makes the sample non-representative of all patients with severe COVID-19, the limited sample size that reduce statistical power, and the observational design that does not allow concluding on causal links. Furthermore, all the included patients were hospitalized during the pandemic first wave and may not be representative of patients who had severe COVID-19 later on. As we tested six hypotheses (i.e., six possible interactions), the significant depression by pain interaction would not have survived correction for multiple tests. In addition, our data contained few biological variables, despite the potential confounding nature of certain biological variables such as the level of cortisol or specific inflammatory cytokines for persistent physical symptoms. We were also not able to take into account any potential past history of depression or other psychiatric disorder, as well as data regarding interpersonal functioning. Furthermore, the sample size did not allow to include much variables in the models. These results should trigger further investigations with larger sample size.

## Conclusion

In this study, we did not find that anxiety at 1 month after hospitalization was associated with persistent physical symptoms at 3 months in patients hospitalized for COVID-19. However, we found that having a greater depression score and a prior pain complaint was nonetheless significantly associated with persistent pain complaint at 3 months, with a similar trend for dyspnea. These intriguing results must be considered with caution, but may warrant focused attention on depressive symptoms following acute COVID-19. Considering the possibility of treating depressive symptoms effectively and early, this association could be a lever in the management of persistent physical symptoms.

## Data Availability Statement

The data analyzed in this study is subject to the following licenses/restrictions: personal health data underlying the findings of our study are not publicly available due to legal reasons related to data privacy protection. Requests to access these datasets should be directed to cedric.lemogne@aphp.fr, jean-sebastien.hulot@aphp.fr.

## Ethics Statement

The study is part of The French COVID Cohort (NCT04262921) that was authorized by the French Ethics Committee (ID RCB:2020-A00256-33). The patients/participants provided their written informed consent to participate in this study.

## The French COVID Study Group

Laurent Abel, Inserm UMR 1163, Paris, France; Claire Andrejak, CHU Amiens, France; François Angoulvant, Hôpital Necker, Paris, France; Delphine Bachelet, Hôpital Bichat, Paris, France; Marie Bartoli, ANRS, Paris, France; Romain Basmaci, Hôpital Louis Mourier, Colombes, France; Sylvie Behilill, Pasteur Institute, Paris, France; Marine Beluze, F-CRIN Partners Platform, Paris, France; Dehbia Benkerrou, Inserm UMR 1136, Paris, France; Krishna Bhavsar, Hôpital Bichat, Paris, France; Lila Bouadma, Hôpital Bichat, Paris, France; Maude Bouscambert, Inserm UMR 1111, Lyon, France; Minerva Cervantes-Gonzalez, Hôpital Bichat, Paris, France; Anissa Chair, Hôpital Bichat, Paris, France; Catherine Chirouze, CHRU Jean Minjoz, Besançon, France; Alexandra Coelho, Inserm UMR 1018, Paris, France; Sandrine Couffin-Cadiergues, Inserm sponsor, Paris, France; Camille Couffignal, Hôpital Bichat, Paris, France; Eric D'ortenzio, ANRS, Paris, France; Marie-Pierre Debray, Hôpital Bichat, Paris, France; Dominique Deplanque, Hôpital Calmette, Lille, France; Diane Descamps, Hôpital Bichat, Paris, France; Mathilde DESVALLÉE, Inserm UMR 1219, Bordeaux, France; Alpha Diallo, ANRS, Paris, France; Alphonsine Diouf, Inserm UMR 1018, Paris, France; Céline Dorival, Inserm UMR 1136, Paris, France; François Dubos, CHU Lille, France; Xavier Duval, Hôpital Bichat, Paris, France; Philippine Eloy, Hôpital Bichat, Paris, France; Vincent Enouf, Pasteur Institute, Paris, France; Hélène Esperou, Inserm sponsor, Paris, France; Marina Esposito-Farese, Hôpital Bichat, Paris, France; Manuel Etienne, CHU Rouen, France; Nathalie Gault, Hôpital Bichat, Paris, France; Alexandre Gaymard, Inserm UMR 1111, Lyon, France; Jade Ghosn, Hôpital Bichat, Paris, France; Tristan Gigante, F-CRIN INI-CRCT, Nancy, France; Morgane Gilg, F-CRIN INI-CRCT, Nancy, France; Jérémie Guedj, Inserm UMR 1137, Paris, France; Alexandre Hoctin, Inserm UMR 1018, Paris, France; Ikram Houas, Inserm sponsor, Paris, France; Isabelle Hoffmann, Hôpital Bichat, Paris, France; Jean-Sébastien Hulot, Hôpital Européen Georges Pompidou, Paris, France; Salma Jaafoura, Inserm sponsor, Paris, France; Ouifiya Kafif, Hôpital Bichat, Paris, France; Florentia Kaguelidou, Hôpital Robert Debré, Paris, France; Sabrina Kali, Hôpital Bichat, Paris, France; Antoine Khalil, Hôpital Bichat, Paris, France; Coralie Khan, Inserm UMR 1219, Bordeaux, France; Cédric Laouénan, Hôpital Bichat, Paris, France; Samira Laribi, Hôpital Bichat, Paris, France; Minh Le, Hôpital Bichat, Paris, France; Quentin Le Hingrat, Hôpital Bichat, Paris, France; Hervé Le Nagard, Inserm UMR 1137, Paris, France; Soizic Le Mestre, ANRS, Paris, France; François-Xavier Lescure, Hôpital Bichat, Paris, France; Sophie Letrou, Hôpital Bichat, Paris, France; Yves Levy, Vaccine Research Institute, VRI; Inserm UMR 955, Créteil, France; Bruno Lina, Inserm UMR 1111, Lyon, France; Guillaume Lingas, Inserm UMR 1137, Paris, France; Jean Christophe Lucet, Hôpital Bichat, Paris, France; Denis Malvy, CHU Bordeaux, France; Marina Mambert, Inserm UMR 1018, Paris, France; France Mentré, Hôpital Bichat, Paris, France; Christelle Paul, ANRS, Paris, France; Amina Meziane, Inserm UMR 1136, Paris, France; Hugo Mouquet, Pasteur Institute, Paris, France; Jimmy Mullaert, Hôpital Bichat, Paris, France; Nadège Neant, Inserm UMR 1137, Paris, France; Marion Noret, RENARCI, Annecy, France; Huong Pham, Hôpital Bichat, Paris, France; Aurélie Papadopoulos, Inserm sponsor, Paris, France; Nathan Peiffer-Smadja, Hôpital Bichat, Paris, France; Ventzislava Petrov-Sanchez, ANRS, Paris, France; Gilles Peytavin, Hôpital Bichat, Paris, France; Valentine Piquard, Hôpital Bichat, Paris, France; Olivier Picone, Hôpital Louis Mourier, Colombes, France; Oriane Puéchal, REACTing, Paris, France; Manuel ROSA-CALATRAVA, Inserm UMR 1111, Lyon, France; Bénédicte Rossignol, F-CRIN INI-CRCT, Nancy, France; Patrick ROSSIGNOL, CHU Nancy, France; Carine ROY, Hôpital Bichat, Paris, France; Marion Schneider, Hôpital Bichat, Paris, France; Richa SU, Hôpital Bichat, Paris, France; Coralie Tardivon, Hôpital Bichat, Paris, France; Marie-Capucine Tellier, Hôpital Bichat, Paris, France; François Téoulé, Inserm UMR 1136, Paris, France; Olivier Terrier, Inserm UMR 1111, Lyon, France; Jean-François Timsit, Hôpital Bichat, Paris, France; Christelle Tual, Inserm CIC-1414, Rennes, France; Sarah Tubiana, Hôpital Bichat, Paris, France; Sylvie van der Werf, Pasteur Institute, Paris, France; Noémie Vanel, Hôpital la Timone, Marseille, France; Aurélie Veislinger, Inserm CIC-1414, Rennes, France; Benoit Visseaux, Hôpital Bichat, Paris, France; Aurélie Wiedemann, Vaccine Research Institute, VRI; Inserm UMR 955, Créteil, France; Yazdan Yazdanpanah, Hôpital Bichat, Paris, France.

## Author Contributions

HB, CG, and CL contributed to conception, design of the study, and wrote sections of the manuscript. DL collected the data. CG performed the statistical analysis. HB wrote the first draft of the manuscript. All authors contributed to manuscript revision, read, and approved the submitted version.

## Funding

This research did not receive any specific grant from funding agencies in the public, commercial, or not-for-profit sectors. Outside the submitted work, J-SH is supported by AP-HP, INSERM, the French National Research Agency (NADHeart ANR-17-CE17-0015-02, PACIFIC ANR-18-CE14-0032-01, CORRECT_LMNA ANR-19-CE17-0013-02), the ERA-Net-CVD (ANR-16-ECVD-0011-03, Clarify project), Fédération Française de Cardiologie, the Fondation pour la Recherche Médicale (EQU201903007852), and by a grant from the Leducq Foundation (18CVD05), and is coordinating a French PIA Project (2018-PSPC-07, PACIFIC-preserved, BPIFrance) and a University Research Federation against heart failure (FHU2019, PREVENT_Heart Failure).

## Conflict of Interest

CL reports personal fees and non-financial support from Janssen-Cilag, Lundbeck, Otsuka Pharmaceutical, and Boehringer Ingelheim in the previous 3 years, outside the submitted work. The APHP, which employs J-SH, has received research grants from Bioserenity, Sanofi, Servier, and Novo Nordisk. J-SH has received speaker, advisory board or consultancy fees from Amgen, Astra Zeneca, Bayer, Bristol-Myers Squibb, Novartis, WeHealth. The remaining authors declare that the research was conducted in the absence of any commercial or financial relationships that could be construed as a potential conflict of interest.

## Publisher's Note

All claims expressed in this article are solely those of the authors and do not necessarily represent those of their affiliated organizations, or those of the publisher, the editors and the reviewers. Any product that may be evaluated in this article, or claim that may be made by its manufacturer, is not guaranteed or endorsed by the publisher.
